# Internet-Based Interventions to Promote Mental Health Help-Seeking in Elite Athletes: An Exploratory Randomized Controlled Trial

**DOI:** 10.2196/jmir.1864

**Published:** 2012-06-29

**Authors:** Amelia Gulliver, Kathleen M Griffiths, Helen Christensen, Andrew Mackinnon, Alison L Calear, Alison Parsons, Kylie Bennett, Philip J Batterham, Rosanna Stanimirovic

**Affiliations:** ^1^Centre for Mental Health ResearchThe Australian National UniversityCanberra, ACTAustralia; ^2^Orygen Youth Health Research CentreUniversity of MelbourneMelbourne, VICAustralia; ^3^Mental Edge ConsultingMelbourne, VICAustralia

**Keywords:** Mental health, help-seeking, elite athletes

## Abstract

**Background:**

Mental disorders are more common in young adults than at any other life stage. Despite this, young people have low rates of seeking professional help for mental health problems. Young elite athletes have less positive attitudes toward seeking help than nonathletes and thus may be particularly unlikely to seek help. Interventions aimed at increasing help-seeking in young elite athletes are warranted.

**Objective:**

To test the feasibility and efficacy of three Internet-based interventions designed to increase mental health help-seeking attitudes, intentions, and behavior in young elite athletes compared with a control condition.

**Methods:**

We conducted a randomized controlled trial (RCT) of three brief fully automated Internet-based mental health help-seeking interventions with 59 young elite athletes recruited online in a closed trial in Australia. The interventions consisted of a mental health literacy and destigmatization condition, a feedback condition providing symptom levels, and a minimal content condition comprising a list of help-seeking resources, compared with a control condition (no intervention). We measured help-seeking attitudes, intentions and behavior using self-assessed surveys. Participation was open to elite athletes regardless of their mental health status or risk of mental illness.

**Results:**

Of 120 athletes initially agreeing to participate, 59 (49%) submitted a preintervention or postintervention survey, or both, and were included in the present study. Adherence was satisfactory, with 48 (81%) participants visiting both weeks of assigned intervention material. None of the interventions yielded a significant increase in help-seeking attitudes, intentions, or behavior relative to control. However, at postintervention, there was a trend toward a greater increase in help-seeking behavior from formal sources for the mental health literacy/destigmatization condition compared with control (*P *= .06). This intervention was also associated with increased depression literacy (*P *= .003, *P *= .005) and anxiety literacy (*P *= .002, *P *= .001) relative to control at postintervention and 3-month follow-up, respectively, and a reduction in depression stigma relative to control at postintervention (*P *= .01, *P *= .12) and anxiety stigma at 3-month follow-up (*P *= .18, *P *= .02). The feedback and help-seeking list interventions did not improve depression or anxiety literacy or decrease stigmatizing attitudes to these conditions. However, the study findings should be treated with caution. Due to recruitment challenges, the achieved sample size fell significantly short of the target size and the study was underpowered. Accordingly, the results should be considered as providing preliminary pilot data only.

**Conclusions:**

This is the first RCT of an Internet-based mental health help-seeking intervention for young elite athletes. The results suggest that brief mental health literacy and destigmatization improves knowledge and may decrease stigma but does not increase help-seeking. However, since the trial was underpowered, a larger trial is warranted.

**Trial Registration:**

2009/373 (www.clinicaltrials.gov ID: NCT00940732), cited at http://www.webcitation.org/5ymsRLy9r.

## Introduction

Mental disorders contribute significantly to worldwide disease burden [1,2]. Depression and anxiety in particular are common, with an estimated prevalence of 6%–18% in high-income countries globally [3-5]. Mental disorders are more common in young adults than at any other stage of the lifespan [3]. Despite this, young people have low rates of seeking professional help for mental health problems [6]. Research indicates that athletes may experience a prevalence of symptoms of depression similar to that of the general population [7] and have less positive attitudes toward seeking help than nonathletes [8]. Since attitudes are thought to influence help-seeking in the general population [6,9,10] and this age group is already at risk, young elite athletes may be particularly vulnerable to not seeking help.

Research has examined the reasons why young people do not seek professional help for mental disorders. A systematic review investigating young people’s perceived barriers to help-seeking found that stigma and embarrassment, problems recognizing symptoms (poor mental health literacy), and a preference for self-reliance were key barriers to seeking help [11]. Factors that might facilitate help-seeking have been comparatively underresearched. However, there has been some evidence that positive past experiences, and social support and encouragement from others may facilitate help-seeking [11]. These factors are said to be effective because they increase mental health literacy and reduce stigma.

Several studies have evaluated interventions designed to improve help-seeking. Previous randomized controlled trials (RCTs) have used information-based methods of targeting help-seeking via video [12], written and verbal information [13-15], and the Internet [16,17]. These interventions have demonstrated an increase in positive attitudes to help-seeking [12-14], willingness to seek help [15], help-seeking beliefs [17], and, less frequently, behavior [16]. Only one trial has been conducted within the sports field. It found that providing information about the benefits of sports psychology in an interview improved athletes’ attitudes to consulting a sports psychologist [13]. There have been no other studies of the efficacy of interventions specifically designed to encourage help-seeking behavior in athletes.

It is not clear from these previous studies what might be the best approach to encouraging help-seeking among youth or particularly elite athletes. Rickwood et al [18] conceptualized seeking professional help as a 4-step process beginning with the individual becoming aware of a problem, then expressing the problem to others, followed by identifying appropriate and accessible sources of help, and then seeking out a health professional and disclosing his or her problems. However, it is not clear which of or how many of these stages are critical to encourage professional help-seeking. Consequently we designed three different interventions, each of which focused on one or more of these stages. The first, a mental health literacy/destigmatization intervention, addressed three of the four stages. First, information was provided to increase the participant’s awareness of a potential problem. Second, the intervention incorporated messages designed to decrease stigma in order to facilitate the expression of a problem. Third, a list of help-seeking sources was provided to assist with the identification of appropriate resources. The second intervention provided feedback about symptoms. The feedback intervention thus focused on the first stage of the Rickwood model to increase awareness of a potential problem. Participants were explicitly provided with information about their levels of symptoms relative to other individuals of a similar age. This intervention also provided information about help-seeking sources. The third intervention was a help-seeking list intervention. This intervention consisted of a list of help-seeking resources to determine whether providing this information alone was sufficient to encourage the participants to seek help. The control condition, where participants received no intervention, was introduced to control for the effect of participating in a research trial and the elapse of time [17].

We hypothesized that, compared with the control group, participants in all three intervention conditions would demonstrate at postintervention and 3-month follow-up improved professional help-seeking attitudes and intentions, and help-seeking from professional sources. Given differences in content and level of intensity of the interventions, we predicted the effects would be strongest in the mental health literacy/destigmatization condition, followed by the feedback condition, and then the help-seeking list condition. Because the help-seeking list was used in all three active interventions, it was possible to compare the relative efficacy of the mental health literacy and feedback components of the first two interventions over and above the effects of the list alone. In addition to differences in help-seeking, we hypothesized that those in the mental health literacy/destigmatization condition would demonstrate increased mental health literacy and decreased stigma compared with those in the control condition.

The three active interventions were delivered through the Internet. Internet-delivered interventions are available 24 hours a day, 7 days a week, can be accessed anonymously, are cost effective [19,20], and can be widely distributed [21,22]. Despite these benefits, only two trials have evaluated the utility of an Internet-based format for encouraging help-seeking. This is the first RCT to examine the feasibility and efficacy of Internet-based help-seeking interventions in elite athletes.

## Methods

The Elite Athlete Mental Health Strategy (TEAMS) project was undertaken in two stages. Stage 1 involved an online survey of elite athletes. This survey incorporated self-report measures of a range of mental health symptoms, and demographic and other participant attributes. Following this, we invited the athletes to participate in stage 2 of the RCT. Inclusion criteria for both stages of the study were being aged 18 years or older and being an elite athlete as defined by their level of competition (Olympic or Paralympic, professional, or state-, national-, or international-level athletes). An implicit inclusion criterion was that the participants should be Internet and computer literate. Ethics approval for the study was granted by both the Australian Institute of Sport (AIS) ethics committee and The Australian National University Human Research Ethics Committee (ANU HREC 2009/373).

### Participants and Recruitment

**Figure 1:**
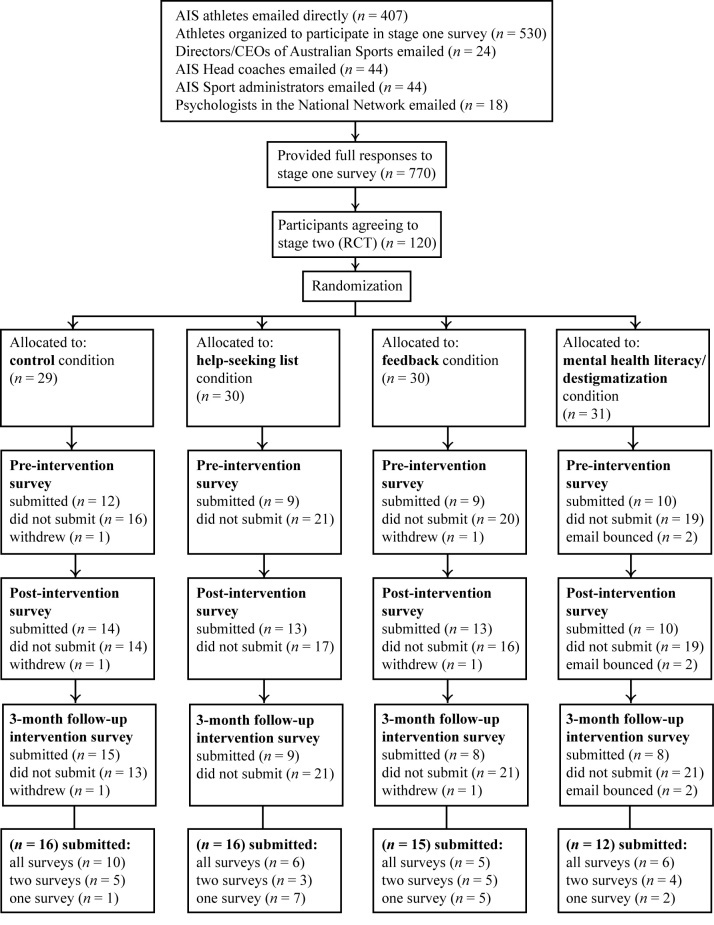
Flow of participants through the trial. AIS = Australian Institute of Sport; CEO = chief executive officer; RCT = randomized controlled trial.

Participants were elite athletes from the AIS and from other national sporting organizations in Australia. They were recruited primarily via emails distributed by the Director of the AIS and through direct recruitment with elite sporting clubs. An initial email invitation containing an embedded link to the information and consent page of the project website was sent by the Director of the AIS in November 2009 to directors and chief executive officers of elite sports organizations around Australia, as well as to all AIS head coaches and administrators, and psychologists from the Australian Psychological Society College of Sport Psychologists. The distribution list was compiled by the AIS. However, this recruitment strategy was of limited effectiveness. Accordingly, we sent a follow-up email to directors and coaches requesting that they distribute the aforementioned emails directly to athletes. However, the number of participants remained low. Therefore, we implemented a third recruitment strategy. This involved sending an initial and a follow-up advertisement-style email from the AIS Director with AIS branding, in both HTML and plain text, directly to all AIS athletes aged 18 years or older (n = 407) during March 2010 (recruitment wave 1). In addition, one organization arranged to send a text message to their athletes’ mobile phones (January 2010) to indicate that they had been sent an email. The final recruitment strategy was to organize a large number of athletes to participate in a group setting at a sports club site using computer facilities. This resulted in a substantial increase in participants (n = 530; see Figure 1) into stage 1 of the project from November 2010 to February 2011 (recruitment wave 2), but a limited number of athletes agreeing to participate in the stage 2 trial.

We offered a random prize draw incentive of an iPhone 3GS (wave 1) and an iPhone 4 (wave 2) to participants who completed the postintervention survey. Incentives have previously been demonstrated to increase participants’ retention rates, training acceptance, and self-assessed effectiveness of Internet-based interventions [23].

Figure 1 presents the flow of participants through the trial. At the conclusion of the stage 1 survey, participants were provided with information about the RCT, and those who agreed to participate provided their email address and were automatically randomly assigned to an intervention arm. Overall, 120 athletes were randomly assigned to the following conditions: control, help-seeking list, feedback, and mental health literacy/destigmatization. A total of 59 participants (16 men, 43 women) completed at least a preintervention or a postintervention survey and were included in the present study results. Participants’ age ranged from 18 to 48 years, and the mean age was 25.5 years (median 24.5).

### Treatment Allocation

In an email message, participants were provided with a link to an anonymous online survey comprising demographic and other mental health measures (stage 1). This survey was located on the AIS-branded Web portal created for the project. One sporting organization arranged for their athletes to complete this initial survey at a set location using laptops (n = 530; see Figure 1). Immediately after completing the stage 1 survey, participants who indicated that they were interested in participating in stage 2 of the project (RCT) were redirected to a webpage on the project website where they could register their email address to be involved in the trial. Participants were then randomly assigned to the different conditions using predefined automated computerized block randomization with a block size of 16. The automated computerized system was set up by researchers not involved in the day-to-day management of the study.

### Data Collection

The intervention and measurement points were spaced 1 week apart and comprised the stage 1 survey (demographics, week 1), preintervention survey (week 2), intervention week 1 (week 3), intervention week 2 (week 4), and postintervention survey (week 5). An automated email was sent to the participants each week with an embedded link to the self-assessed surveys or intervention content. A single reminder email was sent if, after 4 days, participants had not yet accessed their assigned survey or intervention webpage by clicking on the embedded link. Two further follow-up survey emails were sent automatically at 3 and 6 months after the preintervention survey. Each survey took approximately 15–20 minutes to complete. Finally, a debriefing email providing information about the purpose of the study was sent 1 month after the 6-month follow-up email.

### Online Surveys

The information and consent webpage apprised participants of what each stage of the study would involve (including survey time), as well as all requirements listed by the ethics review boards, including when and where data would be securely stored, the investigators and their contact details, and the general purpose of the study. We tested the online surveys by the researchers before deployment and provided them on an open access website; however, we emailed links to this website only to sports managers and elite athletes. Tokens enabled the unique identification of each participant, and each token was embedded into the link to their unique survey. Each page of the survey comprised items from a single measure. Each of the measurement points comprised 10 pages (excluding the stage 1 survey, which comprised 23 pages). All items were mandatory, although participants could review and change their answers before submitting them. In addition, the help-seeking behavior items of the General Help-Seeking Questionnaire (GHSQ) used adaptive questioning, whereby the qualitative items would appear only if the help-seeking sources were selected. Data were automatically captured by the online survey platform, and we used data from all surveys regardless of completeness; thus, participant numbers may differ for each of the outcome variables.

### Interventions

Although participants could not be blinded to their assigned condition, they were informed on the information and consent page that they would be asked to undertake “some online activities” regarding the “usefulness of different types of health information.” This general description reduced the likelihood that participants would be aware of the anticipated effectiveness of their assigned condition.

Intervention content was spaced approximately evenly over 2 weeks and consisted of a series of linear webpages that varied in length depending on the condition type. At the end of each intervention week, the participants were provided with a list of sources of help and resources for mental health problems, which were identical across all intervention conditions at both week 1 and week 2. Sources included face-to-face, telephone, and emergency sources (week 1), and an additional hyperlinked list of online sources for cognitive behavioral therapy and bibliotherapy (week 2). All intervention conditions included an introduction to the week’s activities and a final page congratulating the participant for completing the activities. we made no changes to the intervention content during the trial. The specific content of each of the intervention conditions is described below.

#### Mental Health Literacy/Destigmatization

This condition comprised written material created by one of the researchers (KG) delivered on 34 brief linear webpages. It was designed to increase mental health literacy and decrease stigma, specifically targeting depression and anxiety. The first week contained 19 pages of information about the prevalence and risk of mental disorders; disability and symptoms of depression; a matrix of evidence-based treatments demonstrating the most effective treatments for depression; detailed information about the two most effective depression treatments—antidepressants and cognitive behavioral therapy; a consideration of four myths about depression aimed specifically at reducing stigma; and help-seeking sources (as listed above). The second week was provided in a similar format, this time focusing on generalized anxiety disorder, with 15 pages of brief information about social anxiety, panic, posttraumatic stress, and obsessive compulsive disorders, as well as specific phobia.

#### Feedback

The second condition comprised two interactive quizzes providing tailored feedback to the participant about his or her level of depression (week 1) and anxiety (week 2) using the Goldberg Depression and Anxiety scales [22]. Participants received one of four possible feedback pages corresponding to their symptom score. We derived norms for this feedback from the MoodGYM clinician’s manual [24], which was based on data from a sample of 2404 Australian community-dwelling young adults aged 20–24 years (see [25] for details of sample). It has been suggested that providing feedback is the most efficacious of different types of tailoring mechanisms in implementing behavioral change in smoking cessation [26], and it also appears promising in influencing other health behaviors [27-29]. Thus, we designed this condition to provide an objective measure of depression and anxiety symptoms to allow the participants to assess their need to seek help. The content of this condition was based on the FRAMES framework for effective brief interventions for behavioral change [30]. FRAMES is an acronym for the inclusion of feedback (a form of assessment), responsibility (advising that the behavior is the individual’s responsibility and choice), advice (providing verbal or written advice to change), menu (providing an array of alternative strategies for change), empathy (adopting a warm, reflective, and understanding approach), and self-efficacy (emphasizing the individual’s self-efficacy for change). The condition comprised six pages each week for week 1 (depression) and week 2 (anxiety).

#### Help-Seeking List

This condition comprised three pages each week, including an introduction, help-seeking source page (see above for details), and a congratulatory page.

#### Control

Participants in the control condition received emails to the online measurement surveys only.

### Measures

We collected a large number of demographic and other characteristics and symptoms of mental disorders in the stage 1 survey. However, we present only key, relevant demographic data in the current paper. These included gender, age, highest level of education, level of competition, sports type, psychological distress as measured by the 10-item Kessler Psychological Distress Scale (K10) [31], and prior help-seeking as measured by the prior counseling measure of the GHSQ [32].

#### Primary Outcome Measures

The primary outcome measures were help-seeking attitudes, intentions, and behaviors. Attitudes were measured by the Attitudes Toward Seeking Professional Psychological Help-Short Form (ATSPPH-SF) [33]. The ATSPPH-SF is a 10-item scale measuring attitudes toward seeking psychological help from a professional. Items on the scale include “I would want to get psychological help if I were worried or upset for a long period of time.” Participants indicated their agreement with each statement on a 4-point Likert-type scale ranging from 0 (strongly disagree), 1 (partly disagree), 2 (partly agree), to 3 (strongly agree). This response set was different from the original (strongly disagree and strongly agree should be disagree and agree, respectively) due to a transcription error. Responses were coded from 0 to 3 and summed to calculate a total score, after reverse scoring negatively worded items. Total scores ranged from 0 to 30, with higher scores indicating more positive attitudes to professional psychological help-seeking. Research has previously reported good internal consistency for the ATSPPH-SF in studies of university students (alpha range of .72–.85) [33-36] and medical patients (alpha = .78) [34], as well as adequate validity [33,34] and acceptable test–retest reliability (*r *= .80) [33]. In the present study the internal reliability using Cronbach alpha was .69 (n = 40) at preintervention. Test–retest reliability was moderate between preintervention and 4 weeks postintervention for the control condition (*r* *= *.64; n = 10, *P *= .045).

We measured help-seeking intentions by the intentions scale of the GHSQ [37]. The scale used in the present study consisted of 11 items measuring the respondent’s intentions to seek help for a “personal or emotional” problem. The respondent was first asked “If you were having a personal or emotional problem, how *likely *is it that you would seek help from each of the following sources?” The scale comprised potential sources of formal and informal help (eg, “friend (not related to you)” or “Doctor/GP”) and included an option that they would not seek help from anyone. Each source of help was rated on a 7-point Likert-type scale ranging from 1 (extremely unlikely) to 7 (extremely likely). Item scores ranged from 1 to 7, with higher scores indicating higher intentions to seek help from that particular source. The GHSQ contains two subscales [38]. The first comprises the mean of formal sources (“Doctor/GP” and “Mental Health Professional (eg, school counselor, psychologist, psychiatrist)”, and the second comprises the mean of informal sources (“Intimate partner(eg, significant boyfriend or girlfriend, husband, wife)”, “Friend (not related to you)”, “Mother”, “Father”, and “Other relative/family member”).

The intentions scale of the GHSQ has adequate psychometric properties. Research has previously reported broadly adequate internal consistency in studies of high school students aged 12–19 years for personal or emotional problems (alpha range of .70–.90) [32,39] and university students (alpha = .67) [37], as well as adequate validity [32] and very good test–retest reliability (*r *= .86) [32]. Internal consistency at preintervention in the present study was adequate for formal sources (alpha = .77, n = 37), although lower than ideal for informal sources (alpha = .57, n = 37). Test–retest reliability at preintervention and 4 weeks postintervention for the control condition was low for formal sources (*r *= .42; n = 9, *P *= .26) and high for informal sources (*r *= .91; n = 9, *P *= .001).

Finally, we measured help-seeking behavior by the proportion of participants who reported seeking help from a formal source in the past 3 weeks (since the beginning of the intervention), using the Actual Help-Seeking Questionnaire (AHSQ) [32,40]. This questionnaire consists of 11 items measuring the respondents’ reported help-seeking behavior in the past 2 weeks for a “personal or emotional” problem. Participants were provided a list of help-seeking sources (matched to the list for the intentions scale of the GHSQ reported above [33]) and asked to indicate which, if any, they had “gone to for advice or help in the past 3 weeks.” Participants were also asked to briefly indicate the type of problem they “went to them about.” Participants were also able to indicate that they had not spoken with anyone about their problems or that they had not experienced any problems. As per above, the AHSQ contains two subscales, one based on the formal help-seeking items and the other on the informal items.

#### Secondary Outcome Measures

The secondary outcomes were mental health literacy and stigma.

We assessed mental health literacy specific to depression and anxiety on the Depression Literacy questionnaire (D-Lit) [41] and a new measure, the Anxiety Literacy questionnaire (A-Lit, developed by KG). These measures each consisted of 22 items, with items consisting of a statement assessing the respondents’ knowledge about depression and anxiety, respectively. For each statement, respondents selected what they believed to be the correct response from three possible choices (true, false, or I don’t know). There is a mix of true and false items in each scale. For example, “Loss of confidence and poor self-esteem may be a symptom of depression” (D-Lit; true), and “People with anxiety disorder often hear voices that are not there” (A-Lit; false). Respondents scored 1 point for each correct answer, and total scores ranged from 0 to 22 with higher scores indicating higher literacy for depression (D-Lit) or anxiety (A-Lit).

Psychometric properties are available for the D-Lit. A previous study reported very good internal consistency for the Greek and Italian versions of the D-Lit (alpha = .88 and alpha = .92, respectively) [42], as well as adequate validity and very good test–retest reliability (*r *= .91) [42]. Internal consistency for the D-Lit in the present study was adequate with a Cronbach alpha coefficient of .70 (n = 40). Test–retest reliability for preintervention and 4 weeks postintervention for the control condition was also adequate (*r *= .71; n = 12, *P *= .02). For the A-Lit, internal consistency was acceptable with a Cronbach alpha coefficient of .76 (n = 40), and test–retest reliability for the control condition was very good (*r *= .83; n = 12, *P *= .003).

Similarly, we used two scales to measure personal stigma toward depression or anxiety: the Personal Stigma items of both the Depression Stigma Scale (DSS) [41] and the Generalised Anxiety Stigma Scale (GASS) [43]. The DSS comprises 9 items and the GASS comprises 10 items measuring the respondents’ own stigmatizing attitudes toward depression and anxiety, respectively. Items consist of statements such as “Depression is not a real medical illness” (DSS) and “An anxiety disorder is a sign of personal weakness” (GASS). Respondents indicate their agreement with each statement on a 5-point Likert-type scale ranging from 0 (strongly disagree) to 4 (strongly agree). Responses are summed to calculate a total score, which ranges from 0 to 36 for the DSS, and from 0 to 40 for the GASS. Higher scores on each scale indicate more stigmatizing attitudes toward depression (DSS) or anxiety (GASS). Previous research has reported good internal consistency for the DSS in community studies of adults (alpha range of .76–.82) [41,44], as well as acceptable validity and test–retest reliability (*r *= .66) [41]. In the present study the internal reliability for the DSS was good at preintervention with a Cronbach alpha coefficient of .77 (n = 40). Test–retest reliability for the DSS was high between preintervention and 4 weeks postintervention for the control condition (*r *= .86; n = 12, *P *= .001). The GASS scale has also been shown to have good internal consistency in a community study of adults (alpha = .86) [43], as well as acceptable validity and moderate test–retest reliability (*r *= .58) [43]. In the present study the internal consistency for the GASS was high at preintervention with a Cronbach alpha coefficient of .90 (n = 39). In addition, this sample demonstrated high test–retest reliability for the GASS between preintervention and 4 weeks postintervention for the control condition (*r* *= *.91; n = 12, *P *< .001).

### Statistical Methods

We assessed the study outcomes within an intention-to-treat framework involving the analysis of data across the first three measurement occasions. The effect of the interventions was examined using mixed-models repeated-measures analysis of variance for longitudinal continuous data using SPSS 19.0 for Windows (IBM Corporation, Somers, NY, USA). We used mixed-model logistic regression to analyze longitudinal binary data, using the xtlogit procedure of Stata version 10 for Windows (StataCorp LP, College Station, TX, USA). The within-groups factor was measurement occasion, and the between-groups factor was condition type. Mixed models were used to include all available data, including that from participants who dropped out or did not provide data at pre- or postintervention, under the assumption that data were missing at random [45,46]. The analyses focused on the interactions between the main effects of condition type and measurement occasion. We used planned contrasts to assess change over time between each intervention condition and all other conditions including control. Postintervention contrasts assessed change over time from preintervention to postintervention, and 3-month follow-up contrasts assessed change from preintervention to 3-month follow-up. To evaluate the magnitude of the effects given the small sample size, we estimated between-group effect sizes between each intervention and the control condition with observed means at postintervention and 3-month follow-up using Hedges’ *g *[47] to account for the small sample size. For consistency in interpretation, in scales where a decreasing score indicated an advantage for the intervention group, we reversed the formula so that all effect sizes were positive.

### Power Calculations

The target sample size was 500 participants (125 per condition), which we chose to detect an effect size of 0.25 in help-seeking attitudes between a comparison group and one of the active conditions with 80% power at alpha = .05, assuming a correlation between pre- and postintervention of .5. This sample size was appropriate given the low to moderate effect sizes for help-seeking measures previously detected in RCTs of help-seeking (Cohen *d *range of –0.08 to 0.56) [12-17]. The achieved sample size (n = 59) would have required an effect size of approximately 0.75 standard deviations for 80% power at alpha = .05, and with a correlation between pre- and postintervention of .5. The present study sample was therefore underpowered and the results should be considered as preliminary pilot data.

## Results

Table 1 presents sample characteristics. The age of participants ranged from 18 to 48 years. Participants were from the sports of cricket (n = 14, 24%), soccer (n = 9, 15%), rowing (n = 5, 8%), sailing (n = 4, 7%), field hockey (n = 4, 7%), and multiple (14) other sports (n = 23, 39%). The majority of participants were Olympic athletes (n = 10, 17%) or international-level athletes (n = 28, 48%), while the remainder (n = 21, 36%) were competing at national state, professional, age group international, Paralympic, or age group national state levels.

**Table 1:** Characteristics of participants included in the study.

Characteristic		Intervention arm				Total (n = 59)
		Mental health literacy/destigmatization (n = 12)	Feedback (n = 15)	Help-seeking list (n = 16)	Control (n = 16)	
Age (years), mean (SD)		24.83 (5.32)	25.80 (4.51)	25.44 (7.10)	25.50 (5.68)	25.42 (5.64)
**Gender, n (%)**						
	Male	2 (17%)	5 (33%)	5 (31%)	4 (25%)	16 (27%)
	Female	10 (83%)	10 (67%)	11 (69%)	12 (75%)	43 (73%)
**Highest level of postsecondary/tertiary education, n (%)**						
	Trade/apprenticeship	0 (0%)	0 (0%)	0 (0%)	0 (0%)	0 (0%)
	Other certificate	0 (0%)	3 (20%)	5 (31%)	2 (13%)	10 (17%)
	Associate or undergraduate diploma	0 (0%)	2 (13%)	1 (6%)	0 (0%)	3 (5%)
	Bachelor’s degree	5 (42%)	5 (33%)	5 (31%)	6 (38%)	20 (36%)
	Higher degree	1 (8%)	1 (7%)	0 (0%)	3 (19%)	5 (9%)
	Other	0 (0%)	0 (0%)	0 (0%)	0 (0%)	0 (0%)
	None	6 (50%)	4 (27%)	5 (31%)	5 (31%)	20 (34%)
K10^a ^score, mean (SD)		16.17 (6.55)	16.47 (5.82)	14.00 (1.79)	15.19 (4.76)	15.39 (5.64)
Previously sought help, n (%)		8 (67%)	10 (67%)	9 (56%)	7 (44%)	34 (58%)
Rated helpfulness of the visits, mean (SD)		3.75 (0.89)	3.44 (1.51)^b^	3.11 (1.45)	4.17 (0.98)^b^	3.56 (1.27)

^a ^10-item Kessler Psychological Distress Scale.

^b ^Missing data on this item for one respondent from the feedback condition and one from the control condition.

Table 2 presents the preintervention comparisons between conditions. There were no significant differences between the intervention conditions for any of these variables. In addition, at preintervention there were no significant differences between trial conditions in the proportion of participants who were of Olympic and international (including Paralympic and age group international) level (χ^2^_3 = 0.3,_ *P *= .98).

**Table 2:** Preintervention comparisons between conditions.

Characteristic		Test of significance
Age in years		*F*_3,55 = 0.06, _ *P *= .98
Gender (male/female)		χ^2^_3 = 1.1, _ *P *= .79
Highest level of postsecondary/tertiary education (those with a higher-level education/those without)		χ^2^_3 = 1.8, _ *P *= .65
General psychological distress (K10^a^)		*F*_3,55 = 0.77, _ *P *= .51
Prior counseling experience (yes/no)		χ^2^_3 = 2.2, _ *P *= .55
Rated helpfulness of the visits		*F*_3,28 = 0.91, _ *P *= .45
Help-seeking attitudes (ATSPPH-SF^b^)		*F*_3,36 = 0.59, _ *P *= .63
**Help-seeking intentions (GHSQ** ^c^ **)**		
	Informal sources	*F*_3,33 = 1.24, _ *P *= .31
	Formal sources	*F*_3,33 = 0.70, _ *P *= .56
**Help-seeking behavior (AHSQ** ^d^ **)**		
	Informal sources	χ^2^_3 (n = 40) = 3.7, _ *P *= .33
	Formal sources	χ^2^_3 (n = 40) = 1.0, _ *P *= .85
Depression literacy (D-Lit^e^)		*F*_3,36 = 0.75, _ *P *= .53
Anxiety literacy (A-Lit^f^)		*F*_3,36 = 0.48, _ *P *= .69
Depression stigma (DSS^g^)		*F*_3,36 = 0.80 _ *P *= .50
Anxiety stigma (GASS^h^)		*F*_3,35 = 1.34, _ *P *= .28

^a ^10-item Kessler Psychological Distress Scale.

^b ^Attitudes Toward Seeking Professional Psychological Help-Short Form.

^c ^General Help-Seeking Questionnaire.

^d ^Actual Help-Seeking Questionnaire.

^e ^Depression Literacy questionnaire.

^f ^Anxiety Literacy questionnaire.

^g ^Depression Stigma Scale.

^h ^Generalised Anxiety Stigma Scale.

There was no significant difference between conditions in those missing at both pre- and postintervention from among those who agreed to participate in the trial (N = 120, χ^2^_3 = 1.8,_ *P *= .63). Nor was there a significant difference between conditions in those missing at postintervention (n = 40, χ^2^_3 = 0.9,_ *P *= .92) or at 3-month follow-up (n = 40, χ^2^_3 = 4.6,_ *P *= .22) from among those present at preintervention.

We conducted logistic regression analyses to assess whether there were any significant predictors of agreeing to participate in the trial (n = 120) from those completing the stage 1 survey (N = 770); the completion of any survey (n = 59) from those agreeing to participate (N = 120); and the completion of the postintervention survey (N = 50) from those who completed the preintervention survey (N = 40). Agreeing to participate in the trial was significantly related to gender, with men less likely than women to agree (odds ratio [OR] 0.14, 95% confidence interval [CI] 0.08–0.22, *P *< .001); age, with older participants more likely than younger participants to agree (OR 1.08, 95% CI 1.03–1.13, *P *= .001); prior counseling experience, with those who had *not* received previous counseling more likely to agree (OR 1.74, 95% CI 1.09–2.76, *P *= .02); and general psychological distress, with those with higher K10 scores more likely to agree (OR 1.05, 95% CI 1.01–1.09, *P *= .02). Agreement to participate in the trial was not related to highest level of education. Missingness for both surveys (not submitting either the pre- or postintervention survey after agreeing to participate) was significantly related to gender only, with men more likely than women to be missing (OR 12.90, 95% CI 4.79–34.74, *P *< .001). Missingness for both surveys was not related to condition, age, highest level of education, prior counseling experience, or general psychological distress. Missingness at postintervention (not submitting a postintervention survey) or at 3-month follow-up (not submitting a 3-month follow-up survey) from those present at preintervention was not related to condition, age, gender, posttertiary education, prior counseling experience, or general psychological distress.

### Intervention Adherence

We used unique identifiers (tokens) to track each participant’s use of the intervention materials. Of the 43 study participants from the intervention conditions, 41 (95%) visited at least 1 week’s online program, 35 (81%) visited both weeks’ online programs, and 2 (5%) visited neither. The intervention condition had no effect on the number of online programs visited (*F*_2,39 = 0.13,_ *P *= .88).

### Primary Intervention Effects

Table 3 presents the observed means, standard deviations, and proportions for the primary outcome variables.

**Table 3:** Observed means, standard deviations, and proportions for the primary outcome measures at each measurement occasion for the trial conditions.

Measure		Measurement occasion					
		Preintervention		Postintervention		3-month follow-up	
		n	Data	n	Data	n	Data
**Help-seeking attitudes (ATSPPH-SF** ^a^ **), mean (SD)**							
	Mental health literacy/destigmatization	10	20.30 (3.34)	10	22.40 (3.34)^b^	8	20.63 (4.27)
	Feedback	8	20.67 (3.00)	12	20.67 (4.19)	8	22.63 (4.00)
	Help-seeking list	8	20.22 (2.73)	12	20.92 (3.23)	9	22.44 (3.47)
	Control	11	21.92 (4.10)	14	21.14 (5.26)	15	21.27 (5.16)
**Help-seeking intentions (GHSQ** ^c^ **) – formal sources, mean (SD)**							
	Mental health literacy/destigmatization	10	4.05 (1.52)	10	4.10 (1.29)	8	4.19 (1.60)
	Feedback	8	3.63 (1.85)	12	3.92 (1.22)	8	4.38 (1.66)
	Help-seeking list	8	4.63 (1.22)	12	4.04 (0.92)	9	4.33 (1.32)
	Control	11	4.50 (1.69)	14	3.79 (1.81)	15	4.10 (1.84)
**Help-seeking intentions (GHSQ) – informal sources, mean (SD)**							
	Mental health literacy/destigmatization	10	5.00 (0.92)	10	5.12 (0.81)	8	4.78 (0.93)
	Feedback	8	4.58 (2.05)	12	4.27 (1.49)	8	4.43 (0.88)
	Help-seeking list	8	5.55 (1.18)	12	5.05 (1.29)	9	5.38 (1.09)
	Control	11	5.58 (0.86)	14	5.17 (1.21)	15	5.15 (1.12)
**Help-seeking behavior (GHSQ) – sought formal help, n (%)**							
	Mental health literacy/destigmatization	10	2 (20%)	10	5 (50%)	8	3 (38%)
	Feedback	9	1 (11%)	12	1 (8%)	8	3 (38%)
	Help-seeking list	9	1 (11%)	12	2 (17%)	9	4 (44%)
	Control	12	3 (25%)	14	1 (7%)	15	3 (20%)
**Help-seeking behavior (GHSQ) – sought informal help, n (%)**							
	Mental health literacy/destigmatization	10	6 (60%)	10	7 (70%)	8	6 (75%)
	Feedback	9	8 (89%)	12	7 (58%)	8	6 (75%)
	Help-seeking list	9	5 (56%)	12	8 (67%)	9	7 (78%)
	Control	12	6 (50%)	14	9 (64%)	15	12 (80%)

^a ^Attitudes Toward Seeking Professional Psychological Help-Short Form.

^b ^Significant pattern of change from pre- to postintervention vs feedback, *P* = .04.

^c ^General Help-Seeking Questionnaire.

#### Help-Seeking Attitudes

The overall interaction between condition and measurement occasion for help-seeking attitudes was not significant (*F*_6,68.92 = 1.64,_ *P *= .15). None of the conditions, including the control, had significant changes from pre- to postintervention or from preintervention to 3-month follow-up. However, there was an improvement from pre- to postintervention in attitudes in the mental health literacy/destigmatization condition (*P *= .04) that was significant compared with the feedback condition only, for which the estimated marginal means for attitudes decreased at postintervention. Hedges’ *g *between-group effect sizes at postintervention for the intervention conditions compared with the control condition were 0.28 (95% CI –0.54 to 1.09) for the mental health literacy/destigmatization condition, –0.12 (95% CI –0.91 to 0.67) for the feedback condition, and –0.05 (95% CI –0.82 to 0.72) for the help-seeking list condition. At 3-month follow-up, effect sizes compared with the control for help-seeking attitudes were as follows: mental health literacy/destigmatization condition (*g *= –0.13, 95% CI –0.99 to 0.73), feedback condition (*g *= 0.29, 95% CI –0.58 to 1.15), and help-seeking list condition (*g *= 0.26, 95% CI –0.57 to 1.09).

#### Help-Seeking Intentions

The overall interaction between condition and measurement occasion was not significant for either formal (*F*_3,35.45 = 0.55,_ *P *= .65) or informal help-seeking intentions (*F*_3,26.29 = 2.21,_ *P *= .11). Between-group effect sizes at postintervention for formal and informal intentions between the control condition and the interventions conditions were as follows: mental health literacy/destigmatization condition (formal, *g *= 0.19, 95% CI –0.62 to 1.01; informal, *g *= –0.05, 95% CI –0.86 to 0.76), feedback condition (formal, *g *= 0.08, 95% CI –0.69 to 0.85; informal, *g *= –0.67, 95% CI –1.47 to 0.12), and help-seeking list condition (formal, *g *= 0.17, 95% CI –0.60 to 0.94; informal, *g *= –0.10, 95% CI –0.87 to 0.67). At 3-month follow-up, effect sizes compared with the control for help-seeking intentions were as follows: mental health literacy/destigmatization condition (formal, *g *= 0.05, 95% CI –0.81 to 0.91; informal, *g *= –0.35, 95% CI –1.22 to 0.51), feedback condition (formal, *g *= 0.16, 95% CI –0.70 to 1.02; informal, *g *= –0.69, 95% CI –1.58 to 0.19), and help-seeking list condition (formal, *g *= 0.14, 95% CI –0.69 to 0.97; informal, *g *= 0.21, 95% CI –0.62 to 1.04).

#### Help-Seeking Behavior

The interaction between condition and measurement occasion for help-seeking from formal sources was not significant for any condition compared with the control at postintervention (mental health literacy/destigmatization, OR 57.38, 95% CI 0.85–3868.09, *P *= .06; feedback, OR 5.15, 95% CI 0.04–637.04, *P *= .51; help-seeking list, OR 13.89, 95% CI 0.15–1263.93, *P *= .25) or at 3-month follow-up (mental health literacy/destigmatization, OR 3.48, 95% CI 0.10–122.32, *P *= .49; feedback, OR 9.45, 95% CI 0.18–507.02, *P *= .27; help-seeking list, OR 15.28, 95% CI 0.30–766.56, *P *= .17). Similarly, the overall interaction between condition and measurement occasion for help-seeking from informal sources was not significant for any condition compared with the control at postintervention (mental health literacy/destigmatization, OR 0.74, 95% CI 0.03–19.12, *P *= .86; feedback, OR 0.03, 95% CI 0.00–1.95, *P *= .1; help-seeking list, OR 1.07, 95% CI 0.03–35.22, *P *= .97) or at 3-month follow-up (mental health literacy/destigmatization, OR 0.21, 95% CI 0.01–7.79, *P *= .4; feedback, OR 0.01 95% CI 0.00–1.21, *P *= .06; help-seeking list, OR 0.63, 95% CI 0.01–28.28, *P *= .81).

### Secondary Intervention Effects

Table 4 presents the observed means and standard deviations for the secondary outcome variables, and the significance levels of the planned contrasts comparing change over time between each intervention condition and all other conditions.

**Table 4:** Observed means and standard deviations for the secondary outcome measures at pre- and postintervention for the trial conditions.^a^

Measure		Measurement occasion					
		Preintervention		Postintervention		3-month follow-up	
		n	Mean (SD)	n	Mean (SD)	n	Mean (SD)
**Depression literacy (D-Lit)** ^b^							
	Mental health literacy/destigmatization	10	12.60 (3.63)	10	16.00 (3.50)^c^	7	14.71 (2.36)^d^
	Feedback	9	12.67 (3.50)	11	12.73 (2.94)	7	13.14 (3.76)
	Help-seeking list	9	10.67 (2.29)	12	10.92 (2.54)	9	11.33 (2.83)
	Control	12	12.17 (3.33)	14	12.21 (4.73)	15	11.67 (5.34)
**Anxiety literacy (A-Lit)** ^e^							
	Mental health literacy/destigmatization	10	9.00 (3.53)	10	13.70 (4.88)^f^	7	13.57 (4.16)^g^
	Feedback	9	9.33 (4.15)	11	8.27 (3.23)	7	8.57 (3.95)
	Help-seeking list	9	7.44 (3.84)	12	9.17 (3.19)	9	10.00 (3.64)
	Control	12	8.08 (3.50)	14	9.57 (4.48)	15	10.13 (4.75)
**Depression stigma (DSS)** ^h^							
	Mental health literacy/destigmatization	10	11.50 (2.76)	10	7.50 (4.95)^i^	7	7.71 (3.77)^j^
	Feedback	9	8.44 (6.46)	11	9.82 (5.90)	7	8.57 (5.62)
	Help-seeking list	9	11.11 (5.65)	12	7.58 (3.32)	9	9.56 (4.19)^k^
	Control	12	9.50 (4.32)	14	8.93 (6.39)	15	8.13 (4.70)
**Anxiety stigma (GASS)** ^l^							
	Mental health literacy/destigmatization	10	8.50 (3.27)	10	5.40 (4.77)^m^	7	3.71 (3.35)^n^
	Feedback	8	4.38 (4.10)	11	8.18 (5.38)	7	6.57 (4.50)
	Help-seeking list	9	8.67 (6.21)	12	6.00 (4.22)	9	5.78 (5.07)
	Control	12	6.42 (6.07)	14	5.57 (4.36)	15	6.00 (5.07)

^a ^Significance tests refer to pattern of change.

^b ^Depression Literacy questionnaire.

^c ^Pre- to postintervention vs control, *P *= .003; vs help-seeking, *P *= .004.

^d ^Preintervention to 3-month follow-up vs control, *P *= .005; vs help-seeking, *P *= .04.

^e ^Anxiety Literacy questionnaire.

^f ^Pre- to postintervention vs control, *P *= .002; vs help-seeking, *P *= .003; vs feedback, *P *= .001.

^g ^Preintervention to 3-month follow-up vs control, *P *= .001; vs help-seeking, *P *= .004; vs feedback, *P *< .001.

^h ^Depression Stigma Scale.

^i ^Pre- to postintervention vs control, *P *= .01; vs help-seeking, *P *= .004; vs feedback, *P *= .003.

^j ^Preintervention to 3-month follow-up vs help-seeking, *P *= .002.

^k ^Preintervention to 3-month follow-up vs control, *P *= .04.

^l ^Generalised Anxiety Stigma Scale.

^m ^Pre- to postintervention vs feedback, *P *= .004.

^n ^Preintervention to 3-month follow-up vs control, *P *= .02; vs help-seeking, *P *= .03; vs feedback, *P *= .009.

#### Depression Literacy

The overall interaction between condition and measurement occasion for depression literacy was significant (*F*_6,69.41 = 2.47,_ *P *= .03). Planned contrasts demonstrated in the mental health literacy/destigmatization condition a greater increase in depression literacy from pre- to postintervention than in the control and help-seeking list conditions (see Table 4), and approached significance for the feedback condition (*P *= .05). These effects were maintained at 3-month follow-up. There were no other significant effects. Between-group effect sizes at postintervention compared with control were as follows: mental health literacy/destigmatization condition (*g *= 0.90, 95% CI 0.05–1.75), feedback condition (*g *= 0.13, 95% CI –0.66 to 0.92), and help-seeking list condition (*g *= –0.34, 95% CI –1.11 to 0.44). At 3-month follow-up, effect sizes compared with control for depression literacy were as follows: mental health literacy/destigmatization condition (*g *= 0.66, 95% CI –0.26 to 1.58), feedback condition (*g *= 0.30, 95% CI –0.60 to 1.20), and help-seeking list condition (*g *= –0.07, 95% CI –0.90 to 0.75). Given missing data at postintervention and 3-month follow-up and the lower precision of effect size confidence intervals than of planned contrasts, the effect size confidence intervals include zero even where the contrasts were significant. Patterns were similar for the literacy and stigma outcomes below.

#### Anxiety Literacy

The overall interaction between condition and measurement occasion for anxiety literacy was significant (*F*_6,67.51 = 3.99,_ *P *= .002). Planned contrasts demonstrated in the mental health literacy/destigmatization condition a greater increase in anxiety literacy than in all other conditions at postintervention, and these effects were maintained at 3-month follow-up (see Table 4). There were no other significant effects. Between-group effect sizes at postintervention relative to control were as follows: mental health literacy/destigmatization condition (*g *= 0.90, 95% CI 0.05–1.75), feedback condition (*g *= –0.33, 95% CI –1.12 to 0.47), and help-seeking list condition (*g *= –0.10, 95% CI –0.87 to 0.67). At 3-month follow-up, effect sizes compared with control for anxiety literacy were as follows: mental health literacy/destigmatization condition (*g *= 0.76, 95% CI –0.17 to 1.68), feedback condition (*g *= –0.35, 95% CI –1.25 to 0.56), and help-seeking list condition (*g *= –0.03, 95% CI –0.74 to 0.69).

#### Depression Stigma

The overall interaction between condition and measurement occasion for depression personal stigma was statistically significant (*F*_6,62.22 = 3.20,_ *P *= .008). Planned contrasts demonstrated in the mental health literacy/destigmatization condition a greater decrease in depression stigma than in all other conditions from pre- to postintervention (see Table 4). At 3-month follow-up the effects for the mental health literacy/destigmatization condition were no longer superior for depression stigma compared with any condition, except for the help-seeking list condition, which was associated with greater stigma. There were no other significant effects. Between-group effect sizes at postintervention relative to control were as follows: mental health literacy/destigmatization condition (*g *= 0.25, 95% CI –0.57 to 1.06), feedback condition (*g *= –0.15, 95% CI –0.94 to 0.65), and help-seeking list condition (*g *= 0.26, 95% CI –0.51 to 1.04). At 3-month follow-up, effect sizes compared with control for depression stigma were as follows: mental health literacy/destigmatization condition (*g *= 0.10, 95% CI –0.80 to 0.99), feedback condition (*g *= –0.09, 95% CI –0.99 to 0.81), and help-seeking list condition (*g *= –0.32, 95% CI –1.15 to 0.51).

#### Anxiety Stigma

The overall interaction between condition and measurement occasion for anxiety stigma was significant (*F*_6,65.37 = 2.27,_ *P *= .047). Planned contrasts demonstrated in the mental health literacy/destigmatization condition a greater decrease in anxiety stigma from pre- to postintervention than in the feedback condition only (see Table 4). However, at 3-month follow-up the reduction in anxiety stigma for the mental health literacy/destigmatization condition was significant compared with all conditions. There were no other significant effects. Between-group effect sizes at postintervention relative to control were as follows: mental health literacy/destigmatization condition (*g *= 0.04, 95% CI –0.77 to 0.85), feedback condition (*g *= –0.54, 95% CI –1.35 to 0.26), and help-seeking list condition (*g *= –0.10, 95% CI –0.87 to 0.67). At 3-month follow-up, effect sizes compared with the control for anxiety stigma were as follows: mental health literacy/destigmatization condition (*g *= 0.50, 95% CI –0.41 to 1.41), feedback condition (*g *= 0.12, 95% CI –1.02 to 0.78), and help-seeking list condition (*g *= 0.04, 95% CI –0.78 to 0.87).

## Discussion

### Principal Findings

The present study examined the effects of three brief Internet-based interventions designed to increase help-seeking attitudes, intentions, and behavior in elite athletes. None of the interventions were efficacious in improving mental health help-seeking attitudes, intentions, or behavior relative to the control condition. By contrast, several previous RCTs have demonstrated improvements in help-seeking attitudes for mental health conditions following brief interventions [12-14].

It is likely that the negative finding for help-seeking attitudes in the current study was due to low power, given that it was not feasible to recruit the target sample size. This is supported by our finding that at postintervention the effect size for help-seeking attitudes for the mental health literacy/destigmatization condition (*g *= 0.28) is comparable with those of previous studies, which reported significant intervention effects (between-group effect sizes: *d *range of 0.12–0.34) [12-14]. There was also a trend for an increase in formal help-seeking *behavior *in the mental health literacy/destigmatization condition compared with the control group at postintervention.

It may be relevant that the athletes in the present study had more positive attitudes at preintervention overall (mean score 20.85) than members of a general population sample of a similar age (mean 17.10) [14]. This finding contrasts with the results of a previous research study, which reported that US college athletes had less positive attitudes than their nonathlete peers toward help-seeking for mental health problems from counselors [8]. The higher positive attitudes among athletes in the current sample may have been due to the high proportion of female participants, as women have been found to have more positive attitudes to help-seeking than men [48]. Consistent with this, the female participants in the present sample had more positive help-seeking attitudes than the male participants at preintervention, although this difference was not significant. Further, the significantly lower levels of participation by men in the present study suggest that they may be more difficult to engage with this type of intervention.

The present study found no effect of the interventions on overall help-seeking *intentions*, or on formal or informal help-seeking intentions, for any of the intervention conditions. The negative finding for intentions is consistent with a previous RCT of help-seeking intentions, which used the same measurement scale [17]. It differs somewhat from that of Costin et al [17], who found a significant pre- to postintervention change for help-seeking intentions from formal sources in both the intervention and control conditions. However, again, the present study sample of athletes had relatively high intentions (mean 4.22 out of 7.00) to seek help from formal sources at preintervention. Thus, it is possible that the athlete sample in the current study had less room to improve than participants in the Costin et al study (mean 2.61). Overall, it is encouraging that this sample of athletes had preexisting high levels of intentions to seek help. It suggests that it is important to ensure that those from whom they are likely to seek help are primed and able to deliver evidence-based interventions. It is unclear to what extent the high levels of help-seeking intention were attributable to the relatively high proportion of women in the current study [48]. It was not possible to investigate gender effects in the current study due to the small sample sizes achieved. However, further research should be undertaken to determine whether these interventions exert a differential effect on male and female athletes.

Few RCTs of interventions have investigated or demonstrated an effect on mental health help-seeking behavior. One of the strengths of the design of the present study was the measurement of help-seeking behavior at preintervention to allow comparison between measurement points. In the current study, the change in proportions from pre- to postintervention in those seeking formal help approached significance for the mental health literacy/destigmatization condition. While the sample size is small, these findings suggest that the mental health literacy/destigmatization intervention may be a promising tool for this population, especially given that the trial examined a universal sample not selected for their level of symptomatology. The only previous report of increased help-seeking behavior, by Christensen et al [16], was based on an indicated sample of members of the community with high levels of depressive symptoms.

The interventions did not significantly increase help-seeking behavior from informal sources. However, compared with professional sources, help-seeking behavior from informal sources was high at pre- and postintervention. The fact that athletes were already high seekers of help from informal sources allowed limited room for an increase in this behavior. Moreover, the focus of the interventions was on promoting formal help-seeking.

The mental health literacy/destigmatization condition led to improved depression and anxiety literacy relative to control, an effect that was maintained at 3-month follow-up. It has been argued that increasing mental health literacy in the community is a key means for facilitating help-seeking behavior by individuals and for enabling them to assist others in need [49]. In accordance with this, Han et al [15] concluded that providing psychoeducation (eg, mental health literacy) concerning biological attribution information for depression is an effective method for increasing help-seeking willingness. It is encouraging that the mental health literacy/destigmatization condition in the present study successfully increased knowledge of both depression and anxiety in a brief Internet-based format.

The present study also suggested that the mental health/destigmatization intervention shows promise for decreasing stigma for depression and anxiety. The findings were not as robust as for literacy, with the effect disappearing at 3 months for depression stigma and evident only at 3 month follow up for anxiety stigma. It is possible that stigmatizing beliefs are more difficult to change, given the present study’s stronger results for literacy (*g *range of 0.66–0.90), than stigma (*g *range of 0.04–0.50), although the effects for literacy may be attributable to floor effects. Nevertheless, this preliminary suggestion of a positive effect for stigma is encouraging given that stigma has been implicated as a barrier to help-seeking in young people [11], particularly for depression in adults [50,51].

### Strengths and Limitations

To our knowledge, this is the first study to test the feasibility of targeting help-seeking attitudes, intentions, and behavior in an Internet-based format with elite athletes. Strengths of the study design were that it was universal, targeting not only those in current need of help, but also those who may need to seek help in the future. The focus on online delivery maximized the potential of the intervention to reach a large number and broad range of athletes cost effectively [19] and regardless of geographic location [21]. A further strength of the study was that we were able to track and document a moderately high level of adherence to the intervention. Including active intervention exercises in future interventions may further enhance our ability to track engagement. Finally, the inclusion of three different types of help-seeking interventions provided some preliminary data on the relative effect of each of these methods on help-seeking, stigma, and mental health literacy.

The low achieved sample was the primary limitation of the present study; this decreases the power of the study (increasing chance of type II error). The differing methods of recruitment may have also compromised the external validity of the trial. In addition, the recruitment methods may have resulted in selection bias, in that those who participated in the study may have been generally more interested in the research topic of mental health. As can be seen in Figure 1, athletes were willing to provide a response to the stage 1 survey, whereas only 59 (7.7%) submitted a pre- or a postintervention survey for the RCT. The fact that respondents who agreed to participate in the intervention were not a representative sample of those who completed the initial survey raises the possibility that those who agreed to participate already had positive attitudes toward help-seeking. This may have militated against finding positive effects. The potential for such bias is unlikely to be restricted to the current study, having been noted previously [17]; rather it is a challenge for all studies of help-seeking. Finally, a large majority of the participants were female, which also limits the generalizability of the results. Alternative recruitment strategies targeting low help-seekers, particularly men, are required.

The low recruitment rates may have arisen in part from the inclusion of the lengthy stage 1 survey prior to the invitation to participate in the RCT, with the stage 1 survey inadvertently deterring participation in the RCT. This is an important consideration for future research, especially if the pool of potential participants is small and they have limited time due to demanding schedules as was the case in the present study. In addition, organizing participation by providing computer facilities at the club site markedly increased response rates to the stage 1 survey. The extension of such facilities to the intervention phase might have increased recruitment rates to the trial. Analyses of the factors associated with agreement to participate in the intervention component revealed that certain groups are less amenable to recruitment; there may be value in examining these predictors further with a view to tailoring recruitment strategies to this group. A final limitation is that the changes in the stigma scores might reflect a response bias, whereby the participants in the mental health literacy/destigmatization condition learned how to respond to the stigma items as expected but did not undergo a genuine change in attitudes to depression and anxiety.

### Conclusions

Online interventions that can facilitate mental health help-seeking in targeted populations such as elite athletes have the potential to play an important role in decreasing the prevalence of depression and anxiety disorders in these populations. This exploratory study found that a mental health literacy/destigmatization intervention increased athletes’ knowledge of depression and anxiety disorders, and showed some evidence of reducing their depression and anxiety stigma. It also showed a nonsignificant trend toward improving help-seeking behavior among athletes. Further research is required to evaluate the effect of the mental health literacy/destigmatization intervention employing a sample size that is sufficient to detect a moderate effect size. Further work should be undertaken with athletes and in a range of other settings including schools and workplaces, and including more active components in the intervention to enhance engagement should be considered.

## Abbreviations

AHSQ: Actual Help-Seeking Questionnaire
AIS: Australian Institute of Sport
A-Lit: Anxiety Literacy questionnaire
ATSPPH-SF: Attitudes Toward Seeking Professional Psychological Help-Short Form
CI: confidence interval
D-Lit: Depression Literacy questionnaire
DSS: Depression Stigma Scale
GASS: Generalised Anxiety Stigma Scale
GHSQ: General Help-Seeking Questionnaire
K10: 10-item Kessler Psychological Distress Scale
OR: odds ratio
RCT: randomized controlled trial
TEAMS: The Elite Athlete Mental Health Strategy
